# From social adversity to sympathy for violent radicalization: the role of depression, religiosity and social support

**DOI:** 10.1186/s13690-019-0372-y

**Published:** 2019-10-25

**Authors:** Cécile Rousseau, Ghayda Hassan, Diana Miconi, Vanessa Lecompte, Abdelwahed Mekki-Berrada, Habib El Hage, Youssef Oulhote

**Affiliations:** 1 Division of Social and Cultural Psychiatry, SHERPA Research Center, McGill University, CIUSSS Centre-Ouest-de-l’lle-de-Montreal, CSSS de la Montagne (Park Extension), 7085 Hutchison, Suite 204.2, Montreal, QC H3N 1Y9 Canada; 2 UQAM, J.-A.-DeSeve Pavillon, Suite DS-1900, 320 Sainte-Catherine St. E., Montreal, QC H2X 1L7 Canada; 3 CLSC Parc Extension, McGill University, 7085 Hutchison, Room 304-AA, Montreal, QC H3N 1Y9 Canada; 4 McGill University, James Administration Building, 845 Sherbrooke St. W., Room 400, Montreal, QC H3A 0G4 Canada; 5 Anthropology Department, Laval University, Charles-De Koninck Pavillon, Suite 6403, 1030 Sciences humaines Av., Quebec City, QC G1V 0A6 Canada; 6 Rosemont College, C.P. 8888, succursale Centre-ville, Montreal, QC H3C 3P8 Canada; 7 School of Public health and Health Sciences, University of Massachusetts at Amherst, Arnold House, 716 North Pleasant Street, Amherst, MA 01003 Canada

**Keywords:** Violent radicalization, Depressive disorders, Discrimination, Health policy, Pediatrics, Psychiatry, Psychology, Public health

## Abstract

**Background:**

Discrepancies among studies suggest that the relation between social adversity and sympathy for violent radicalization (SVR) is multifaceted and may differ according to social context. This paper examines the role of depression, religiosity and social support in the relation between social adversity (i.e., discrimination and exposure to violence) and SVR among college students in Quebec, Canada.

**Methods:**

A total of 1894 students responded to an online questionnaire posted on the internet of eight colleges. Multilevel analyses were first conducted to account for the clustered nature of the data, followed by mediation and moderation analyses.

**Results:**

First generation migrants reported less SVR than second generation youth and non-immigrants. The mediating and/or moderating role of depression, religiosity and social support was examined through causal inference models. Depression mediated the relation between social adversity and SVR, with depression scores accounting for 47% and 25% of the total effect between discrimination and exposure to violence and SVR scores, respectively. Religiosity and social support moderated the association between social adversity and SVR.

**Conclusions:**

These results suggest that prevention programs should consider violent radicalization as a systemic issue which involves both minorities and the majority, although the specific balance between risk and protective factors may be influenced by local dynamics. They also question intervention measures targeting specifically migrants or ethno-cultural communities because of the risk of increasing profiling and stigmatization. Prevention programs should prioritize decreasing discrimination in colleges, as well as the provision of psychosocial support to depressed youth who experience social adversity.

## Background

Although a social phenomenon, violent radicalization is increasingly considered as a public health concern [1, 2] because of its consequences on well-being, and of its relation with mental health issues. The different paths leading to violent radicalization have been associated with macro-level factors, such as national and international policies; meso-level factors, such as social grievances and social networks; and micro-level factors, such as social adversity and psychological issues [3–6].

In this paper, social adversity is defined specifically in terms of perceived discrimination and exposure to violence, which represent two crucial risk factors for violent radicalization [7, 8]. The available empirical evidence shows that different forms of discrimination are related with an increased support for radical actions [4, 9, 10] and an increase in terrorist attacks [11, 12]. Trauma and post traumatic symptomatology, which involve different degrees of exposure to violence, have also been associated with more radicalism in young Somali refugees [13]. However, there is a paucity of studies investigating how discrimination and exposure to violence are linked to Sympathy for Violent Radicalization (SVR) in both majority and minority groups in the general population. Given the growing number of homegrown young men who embrace violent radicalization processes, as well as the present growth in the number of terrorist attacks, hate crimes and xenophobic incidents worldwide [14–16], the study of the social determinants of SVR represents a top priority in a public health perspective [7, 17]. Of importance, evidence on potential risks and protective factors able to buffer the negative consequences of social adversity on risk of SVR is warranted to inform prevention and intervention actions. Although systematic literature reviews have emphasized that SVR cannot be equated with actual involvement in violent extremism, evidence suggests it is an indicator of the relative attraction exerted by extremist discourses [18].

Overall, systematic literature reviews have insisted on the absence of a specific psychological profile of radicalized individuals [18], supporting the need to focus on a complex interaction of individual, local and social variables. In light with this mounting evidence, the present paper adopts an ecological perspective [1] and focuses on the complex interplay among several variables which have been linked to SVR, namely social adversity, depression, religiosity and social support, as to inform prevention and intervention programming in a public health approach.

### Mediating and moderating factors in the association between social adversity and SVR

In the UK, Bhui et al., [19, 20] found that, regardless of social adversity, depressive symptoms were associated with more SVR in a sample of Muslim Pakistani and Bangladeshi family-origin adults. The authors did not find a mediating role of depression in the relation between life adverse events and SVR, suggesting an independent relation between depression and aggression. However, the life events they considered were all personal (e.g., loss of a relative/friend) and did not tackle exposure to violence. Their findings raised the hypothesis that the social upheaval around violent radicalization may sometimes influence the manifestation of hopelessness and despair and eventually channel them through these new forms of expression. However, in contradiction with this hypothesis, Coid and al [21]., reported more depression in young men with neutral or undecided views, than with those who supported extremist beliefs. These discrepancies between studies suggest that the role of depression in relation to SVR as an independent factor, or as a mediating or moderating factor of social adversity, may vary across different social groups and contexts, and certainly requires further study [7, 22].

Another controversial variable which has been associated with violent radicalization is religiosity, which indicates how important one’s religion is for the self and one’s level of religious involvement in terms of frequency of attendance to organized and non-organized religious activities [23]. Although we witness on a daily basis a public rhetoric which associates religion, especially Islam, with violent radicalization [7, 16], empirical evidence in support of this relation is still lacking. On the contrary, religiosity is a documented protective factor in the relation between life stressors and delinquency in both majority and minority samples [24, 25], and preliminary evidence suggests it could also play a role in buffering the expected relation between social adversity and SVR [21, 26]. However, it is also possible that social adversity may weaken one’s religiosity, thus attributing to religiosity a mediational role in links with SVR [27, 28]. Yet, the potential moderating or mediating role of religiosity in the link between social adversity and SVR needs to be further explored.

As regards social support, extensive evidence has highlighted that it is negatively associated with depression and suicidal risk [29, 30], and can buffer the negative consequences of contextual and life adversities on one’s psycho-social adjustment, representing a well-known protective factor for mental health [25, 31, 32]. In addition, preliminary evidence suggests that the possibility to count on a solid social network and on social support reduces the risks of becoming involved in violent radicalization processes [13, 33]. However, no study so far has investigated in a same empirical model the contributions of depression, religiosity and social support in the expected association between social adversity and SVR.

#### The present study

The present study adopts a public health and ecological framework to investigate the mediators and moderators of the relation between social adversity and SVR in a majority-minority sample of Quebec college students. We hypothesize that: 1) social adversity (i.e., discrimination and exposure to violence) would be associated with more SVR; 2) depression would mediate/moderate the relationship of social adversity with SVR; 3) religiosity would mediate/moderate the relationship of social adversity with SVR, and 4) social support would moderate the relationship between social adversity and SVR, acting as a buffer of adversity.

## Method

### Participants

College students were invited to take part in this study in 2016, in 8 Colleges located in different areas of Quebec, Canada. In Quebec, colleges (known as Cégeps) are public educational institutions placed between high school and university. Their purpose is to prepare youth for university or for technical careers. These types of schools provide two to three year pre-university programs and vocational career programs to younger students (starting from age 16) as well as older professionals. Participants were included in the study if they were registered as a full-time student in one of the participating Colleges. Students participated by completing an online questionnaire that was uploaded on each College’s intranet portal and remained online for a month. Response rate varied greatly between the 8 colleges, ranging from 2 to 19%. A total of 1894 participants provided incomplete data, and full data on the outcome of interest was available for 1190 participants. Participants completed the questionnaire in either French or English. The study protocol and procedures were approved by the Ethics Committee of the Centre Intégré Universitaire de Santé et de Services Sociaux du Centre-Ouest-de-l’Île-de-Montréal (CIUSSS-CODIM, protocol #16–258–2017-606) as well as by the research ethic boards of each institution. Participants gave electronic informed consent before completing the online questionnaire. Given that the research project was considered to involve minimum risk for the participating minors (i.e., 16 years old or older), parental consent was not required, in line with the Section 21 of the Quebec Civil code. Participant socio-demographic characteristics are presented in Table 1.

**Table 1:** Sociodemographic characteristics of participants

Characteristics	n (%)	
	Included (*n* = 1190)	All (*n* = 1894)
Age		
16–18 years	435 (37%)	696 (37%)
19–21 years	430 (36%)	685 (36%)
22–24 years	120 (10%)	196 (11%)
≥ 25 years	203 (17%)	309 (16%)
Missing	2	8
Sex		
Men	351 (30%)	606 (32%)
Women	839 (70%)	1288 (68%)
Language		
French	832 (70%)	1288 (69%)
English	63 (5%)	97 (5%)
Both	295 (25%)	472 (26%)
Missing	0	37
Immigration status		
≥ 3rd generation	771 (66%)	1189 (64%)
2nd generation	191 (17%)	304 (16%)
1st generation	202 (18%)	366 (20%)
Missing	26	35
Religion		
None	646 (57%)	1008 (56%)
Christianism	388 (34%)	618 (34%)
Islam	63 (6%)	126 (7%)
Other	31 (3%)	47 (2%)
Missing	62	95
Experience of violence		
≥ 1 experience of violence	522 (44%)	738 (46%)
Missing	8	300
Discrimination		
≥ 1 experience of discrimination	427 (38%)	568 (37%)
School setting	282 (24%)	375 (24%)
Missing	30	350
Depression (cutoff)		
≤ 1.75	643 (61%)	694 (62%)
> 1.75	406 (39%)	434 (38%)
Missing	141	766
Anxiety (cutoff)		
≤ 1.75	780 (71%)	853 (72%)
> 1.75	319 (29%)	339 (28%)
Missing	91	702
Social Support (median)		
≤ 22	658 (55%)	707 (55%)
> 22	530 (45%)	568 (45%)
Missing	2	619
Colleges		
College 1	139 (12%)	213 (12%)
College 2	378 (32%)	598 (32%)
College 3	23 (2%)	41 (2%)
College 4	238 (20%)	357 (19%)
College 5	46 (4%)	66 (4%)
College 6	172 (14%)	268 (15%)
College 7	115 (10%)	187 (10%)
College 8	67 (6%)	104 (6%)
Missing	12	60

### Measures

#### Social adversity

Students’ exposure to violence was investigated via three questions used in the Enquête Santé Québec on Cultural Communities [34]. Participants were asked (yes/no response format) whether: 1) they witnessed or experienced acts of violence in relation to a social and/or political context; 2) they had a personal experience of persecution, and 3) they witnessed or experienced violent events involving someone close (e.g., family, friend). Participants who answered yes to at least one of the questions were categorized as exposed to violence. The Perceived Discrimination scale [35] is a self-report questionnaire that documents the experience of structural discrimination in eight domains of life (i.e., employment, workplace, housing, academic, public services, health services, social services and justice system). Participants are asked if they experienced discrimination in any of the selected eight domains of life and are invited to answer in a dichotomous format (i.e., yes/no response). According to their answers, students were assigned to one of two groups: 1) those who experienced discrimination in at least one of the domains (i.e., at least one yes response), and 2) those who did not report discrimination in any domain (i.e., all no responses). This questionnaire also yields a continuous score for different types of explicit (e.g. racist insults, threats or aggression) and implicit (e.g., passive exclusion from a group) discriminatory events. Participants identify the frequency of occurrence of each event on a scale from 1 (never) to 6 (constantly), with scores ranging from 11 to 66. In this study, the Cronbach alpha for the total score is .87.

#### Depression and anxiety

The Hopkins Symptom Checklist-25 (HSCL-25) is a self-report questionnaire aimed at screening for levels of anxiety and depression. Items are rated on a Likert scale from 1 (not at all) to 4 (extremely), and a total score is obtained by computing the mean of all items. The clinical cut-off is set at 1.75 (score range from 1 to 4). The HSCL-25’s psychometric qualities and transcultural validity have been well established among different cultural groups [36–39]. In this study, the Cronbach alpha for the total score is .94, for the depression score .92, and .87 for the anxiety score.

#### Religiosity

The revised Religious Orientation Scale [23] aimed to document intrinsic and extrinsic religious orientation. It is a 11-item measure marked on a 5-point scale, with higher scores indicating higher religiosity (score range 11–55). Psychometric properties are good with diverse populations. In this study, the total score was used (α = .90).

#### Social support

The Multidimensional Scale of Perceived Social Support (MPSS) [40], a self-report instrument with good transcultural psychometric properties [41], was used to assess perceived social support from family and friends (4 items). The response options are scored from 1 (very strongly disagree) to 7 (very strongly agree). Scores on all items are summed to obtain a composite scale score (i.e. global perceived social support) ranging from 4 to 28, with higher scores indicating a higher perceived social support. In this study, the Cronbach alpha for the global score is .79.

#### Sympathy for violent radicalization

A modified version of the Sympathies for Radicalization scale (SyfoR) [20] rates participants’ degree of sympathy or condemnation of nine acts of protest ranging from nonviolent (e.g. take part in non-violent political protests) to progressively more extreme/terrorist acts (e.g. use of bombs or weapons to fight against injustices). The participant answers on a 7-point Likert scale ranging from (1 = completely condemn to 7 = completely sympathize, 0 = refuse to answer) with a higher score meaning greater sympathies for violent radicalization. A total score (α = .86, range 8–56) of sympathy for radicalization was used in this study (excluding the non-violent protest item).

The Radicalism Intention Scale (RIS) is a subscale of the Activism and Radicalism Intention Scales (ARIS) developed and validated by Moskalenko and McCauley [42]. The RIS assesses an individual’s willingness to support illegal and violent behavior in the name of one’s group or organization. It is composed of four items rated on a 7-point Likert scale ranging from 1 = disagree completely to 7 = completely agree; with higher total score indicating more support to violent radicalization. The total score (α = .82, range 4–28) was used in this study.

### Statistical analyses

For all analyses, discrimination, depression, and religiosity scores were standardized, therefore, allowing for inference of the effect of a one Standard Deviation (SD) increase in the exposure on SVR scores. Analyses estimating the effects of discrimination on SVR scores were conducted using both standardized discrimination scores and the dichotomous variable, i.e. whether or not the students have experienced structural discrimination at least in one of the eight domains. We used χ2 tests, t tests, or ANOVA to examine univariate associations between the discrimination, Exposure to Violence (ExV), and SVR scores and students’ socio-demographic characteristics.

We used directed acyclic graphs (DAGs) to identify the minimum set of confounders sufficient to estimate the effects of discrimination and exposure of violence on the SVR scores. Among the list of measured characteristics, age, gender, immigration status, religion, and language were inferred from the DAG and we therefore included these variables in the models.

First, we used multilevel analyses to estimate the total effect of ExV and discrimination on SVR scores to account for the clustered nature of data within colleges. Students from the same institution are expected to respond more similarly than students from different institutions as there are other institutional factors that can impact the response. Therefore, our statistical analysis accounts for this intra-institution correlation by using multi-level regression analyses. Next, we assessed whether depression and religiosity levels, as well as social support, moderated the ExV and discrimination effects on SVR scores (i.e., if the effects differed for high vs. low moderator levels) using cross-product terms in the models. For these interaction analyses, depression scores were dichotomized at the clinical cutoff of 1.75, religiosity scores were dichotomized at the median (median = 15, range: 0–55), and social support scores were dichotomized at the median (median = 22, range: 1–28). Finally, we determined the extent to which depression and religiosity may mediate the effects of ExV and discrimination on SVR scores. We ran separate mediation analyses for each of the mediators. The mediation analyses yielded estimates of the direct effect, or the effects not attributable to depression or religiosity, as well as an indirect effect (i.e., the proportion of the total effects we can attribute to depression or religiosity). The indirect effect was used to calculate the proportion of the effect attributed to mediators.

We used Monte Carlo approximation based on the asymptotic sampling distribution [43] to compute confidence intervals in mediation analyses. Since traditional approaches to mediation analyses proposed by Baron and Kenny [44] only apply in specific cases of linear regression for both the mediator and the outcome models with no exposure-mediator interaction, we choose to apply mediation analyses within the potential outcome framework to relax these assumptions. Causal inference methods for mediation analysis are an extension of the traditional approach, developed to better address these main limitations, in addition to a third limitation pertaining to potential intermediate confounding. They allow for effect decomposition by defining direct and indirect effects that are not model specific within the counterfactual framework [45]. To direct and indirect effects under the sequential ignorability assumption [45], let *M(a)* denote the potential value of the mediator of interest under the exposure status *A = a.* Let *Y(a,m)* denote the potential outcome that would result if the exposure *A = a* and the mediator *M = m*, respectively. For simplicity, we illustrate the estimates using a binary exposure and mediator taking values of 0 and 1. Under this framework, the Total Effect (TE) can be expressed as follows: *TE* = *E*[Y(A = 1, M(1)) − Y(A = 0, M(0))]. We can therefore decompose this total effect into two components. First, the Average Causal Mediation Effect (ACME): *ACME* = *E*[Y(A = a, M(1)) − Y(A = a, M(0))] [46, 47] for each exposure status *a = 0, 1*. This quantity corresponds to the change in Y that would occur if one changes the mediator from the value that would be realized under the control condition, *M (0)*, to the value that would be observed under the exposure condition *M(1)*, while holding the exposure status at *A = a*. All other causal mechanisms (average direct effect [ADE]) can be represented by the direct effects of the exposure as: *ADE* = *E*[Y(A = 1, M(a)) − Y(A = 0, M(a))] for each exposure status *a = 0, 1*. This quantity represents the direct effect of the treatment A on the outcome Y, while holding the level of the mediator M constant at the level that would be realized under the exposure condition. Together, ACME and ADE sum up to the total effect. In practice, the outcome is modeled as a function of the mediator, the exposure, and the pre-exposure covariates. The models can be linear, nonlinear, or semiparametric. Based on the mediator model, we generate two sets of predictions for the mediator, one under the exposure status and the other under the control. For example, for ExV as the exposure and depression scores as the potential mediator, this would correspond to predicted levels of depression after Experiencing violence (ExV = 1) or not (ExV = 0). For the next step, the outcome model is used to make potential outcome predictions. Suppose that we are interested in estimating the ACME under the *ExV = 1*, i.e., ACME (1). First, the outcome (SVR score) is predicted under the treatment (*ExV = 1)* using the value of depression scores predicted in the treatment condition *M (ExV = 1)*. Second, the SVR score is predicted under the treatment condition (*ExV = 1)* but now uses the depression scores predicted from the control condition *M (ExV = 0)*. The ACME is then computed as the average difference between the SVR score predictions using the two different values of depression scores.

In a final analysis, and for policy intervention purposes, we also present results for a counterfactual conditional direct effect (CDE) that represents the effect of the exposure (ExV and discrimination) on SVR scores if we were to intervene on the mediator (depression scores or religiosity) and hold it to a specific value (e.g. for depression if we were to intervene on depression and hold depression scores for all students below the clinical cutoff of 1.75). For this analysis, depression scores were dichotomized at the clinical cutoff as depressed or not depressed (1 if depression score > 1.75 and 0 if ≤1.75), the conditional direct effect representing the effect of exposures on the SVR scores when intervening on depression scores to keep all the students below the clinical cutoff is therefore: *CDM*(0) = *E*[*Y*(*A* = 1, *M* = 0) − *Y*(*A* = 0, *M* = 0)]. This is an important estimand since it informs about the direct effect when we intervene on the mediator.

All analyses were performed on a complete case basis with no imputation of missing data. The threshold for statistical significance was set to 0.05 (two-sided tests). We used the mediation package (Tingley et al., 2014) in R (R Foundation for Statistical Computing, Vienna, Austria).

## Results

SVR scores ranged between 0 and 63 with a mean of 22.7 (see Table 2).

**Table 2:** Descriptive statistics of study variables

Characteristics	Experience of violence			Discrimination (continuous score)			Sympathy for Violent Radicalization (SVR; SyfoR)		
	n	Prevalence	*P*-value	n	Mean (SE)	*P*-value	n	Mean (SE)	*P*-value
Age	1178		0.44	1117		< 0.001	1188		0.03
16–18 years	431	44.8%		401	15.9 (0.4)		435	23.1 (0.4)	
19–21 years	428	47.7%		403	16.5 (0.4)		430	23.8 (0.5)	
22–24 years	119	51.3%		113	16.9 (0.8)		120	23.8 (0.9)	
≥ 25 years	200	50.5%		200	18.7 (0.6)		203	19.2 (0.7)	
Sex	1180		0.41	1116		0.85	1190		< 0.001
Men	347	45.5%		330	16.8 (0.4)		351	25.0 (0.6)	
Women	833	48.1%		768	16.7 (0.3)		839	21.8 (0.3)	
Language	1180		0.01	1116		0.01	1190		0.3
French	826	44.9%		830	16.2 (0.2)		832	22.7 (0.3)	
English	63	61.9%		24	17.9 (1.6)		63	24.6 (1.3)	
Both	291	51.2%		262	18.1 (0.5)		295	22.6 (0.6)	
Immigration status	1154		< 0.001	1095		0.02	1164		< 0.001
≥ 3rd generation	766	41.4%		747	15.6 (0.2)		771	23.2 (0.3)	
2nd generation	188	61.7%		160	18.2 (0.7)		191	24.5 (0.8)	
1st generation	200	55.0%		188	18.6 (0.6)		202	19.8 (0.7)	
Religion	1118		0.02	1056		0.01	1128		< 0.001
None	641	41.3%		612	16.1 (0.3)		646	24.5 (0.4)	
Christianism	384	46.1%		364	16.7 (0.4)		388	19.9 (0.4)	
Islam	62	61.3%		55	19.7 (1.3)		63	20.9 (1.1)	
Other	31	61.3%		25	18.5 (2.1)		31	19.5 (1.9)	
Depression (cutoff)	1039		< 0.001	1049		< 0.001	1049		0.005
≤ 1.75	636	36.8%		643	14.8 (0.2)		643	21.9 (0.4)	
> 1.75	403	59.3%		406	19.8 (0.4)		406	23.7 (0.5)	
Religiosity (median)	1065		0.07	1071		0.004	1074		< 0.001
≤ 15	539	43.2%		544	16.0 (0.3)		544	23.8 (0.4)	
> 15	526	48.9%		527	17.3 (0.3)		530	21.6 (0.4)	
Social Support (median)	1178		< 0.001	1114		< 0.001	1188		0.03
≤ 22	653	54.4%		603	18.2 (0.3)		658	23.3 (0.4)	
> 22	525	38.5%		511	14.9 (0.3)		530	22.0 (0.4)	
Colleges	1168		0.006	1104		0.46	1178		0.04
1	137	42.3%		136	17.0 (0.6)		139	22.9 (0.8)	
2	374	47.3%		378	16.3 (0.4)		378	21.9 (0.4)	
3	23	43.5%		23	15.0 (1.0)		23	22.9 (1.9)	
4	236	40.2%		233	16.6 (0.5)		238	22.8 (0.6)	
5	46	54.3%		46	16.9 (1.1)		46	19.5 (1.2)	
6	172	47.1%		165	17.1 (0.6)		172	23.0 (0.9)	
7	113	59.3%		115	18.0 (0.7)		115	24.8 (1.1)	
8	67	62.7%		8	18.5 (2.6)		67	24.6 (1.3)	
Total	1182	47.4%		1118	16.7		1192	22.7	

Results are reported separately for each study variable. *P*-value of the effect of each socio-demographic variable and moderating/mediating variable on each study variable is reported

SVR scores were significantly higher among boys, students between 19 and 24 years of age, students declaring no religion, and in students from second (at least one parent born outside of Canada) and third (both parents born in Canada) generation. Additionally, SVR scores were significantly higher among students reporting higher depression scores, lower religiosity, and lower social support (Table 2).

School was the most frequently reported place where perceived discrimination was experienced (24%). Students experiencing discrimination in the school setting mostly reported ambiguous forms of discrimination. For example, 22% of them reported that they perceived at least once a week that people acted as if they were better than them, and 16% felt people acted frequently as if they were not smart.

### Associations between ExV, discrimination, and SVR scores

After adjustment for age, sex, religion, immigration status, and language while allowing for random intercepts for colleges, ExV was significantly associated with 2.6 points (95% Confidence Interval (CI): 1.5, 3.7) higher SVR scores. Likewise, a one SD increase in discrimination scores was significantly associated with 0.97 points (95% CI: 0.4, 1.6) higher SVR scores. When analyzing discrimination events dichotomously, students having reported at least one discrimination event (for the eight domains) had significantly higher SVR scores (β = 2.2; 95% CI: 0.9, 3.4).

### Moderation and mediation analyses

In moderation (Interaction) analyses, depression scores did not appear to moderate the association between discrimination, and ExV and SVR scores (Table 3).

**Table 3:** Results from moderation (Interaction) analyses (*n* = 1190)

Exposure	Moderator	Low exposure to moderator		High exposure to moderator		P-interaction
		Estimate	95% CI	Estimate	95% CI	
Discrimination (continuous score)	Depression (≤1.75; > 1.75)	0.67	−0.29, 1.63	0.69	−0.12, 1.51	0.97
	Religiosity (≤15; > 15)	1.72	0.90, 2.54	0.29	−0.53, 1.12	0.02
	Social support (≤22; > 22)	1.13	0.42, 1.84	0.47	−0.61, 1.54	0.31
Discrimination (Dichotomous; < / ≥ 1 event)	Depression (≤1.75; > 1.75)	1.54	−0.15, 3.23	1.06	−0.91, 3.03	0.71
	Religiosity (≤15; > 15)	1.84	0.09, 3.60	2.58	0.77, 4.39	0.56
	Social support (≤22; > 22)	3.05	1.45, 4.65	0.74	−1.14, 2.62	0.06
Experience of Violence	Depression (≤1.75; > 1.75)	2.67	1.15, 4.20	1.03	−0.87, 2.94	0.20
	Religiosity (≤15; > 15)	3.73	2.10, 5.35	1.79	0.11, 3.47	0.09
	Social support (≤22; > 22)	2.70	1.20, 4.20	2.43	0.70, 4.15	0.82

However, analyses for religiosity showed significant interactions with discrimination and ExV in the association with SVR scores. For instance, the association between discrimination scores and SVR scores was significantly (*p* = 0.02) lower in students with high religiosity scores (β for 1-SD increase in discrimination scores = 0.29; 95% CI: − 0.53, 1.12) compared to students with low religiosity scores (β = 1.72; 95% CI: 0.90, 2.54). A similar trend (*p* = 0.09) was observed for ExV with an association with SVR scores lower in students with high religiosity scores (β = 1.79; 95% CI: 0.11, 3.47) compared to students with low religiosity scores (β = 3.73; 95% CI: 2.10, 5.35). Finally, Social support scores modified significantly the association between discrimination events (dichotomous) and SVR scores, with a stronger association in students with low social support (β = 3.05; 95% CI: 1.45, 4.65) compared to students with high social support (β = 0.74; 95% CI: − 1.14, 2.62).

**Fig. 1:**
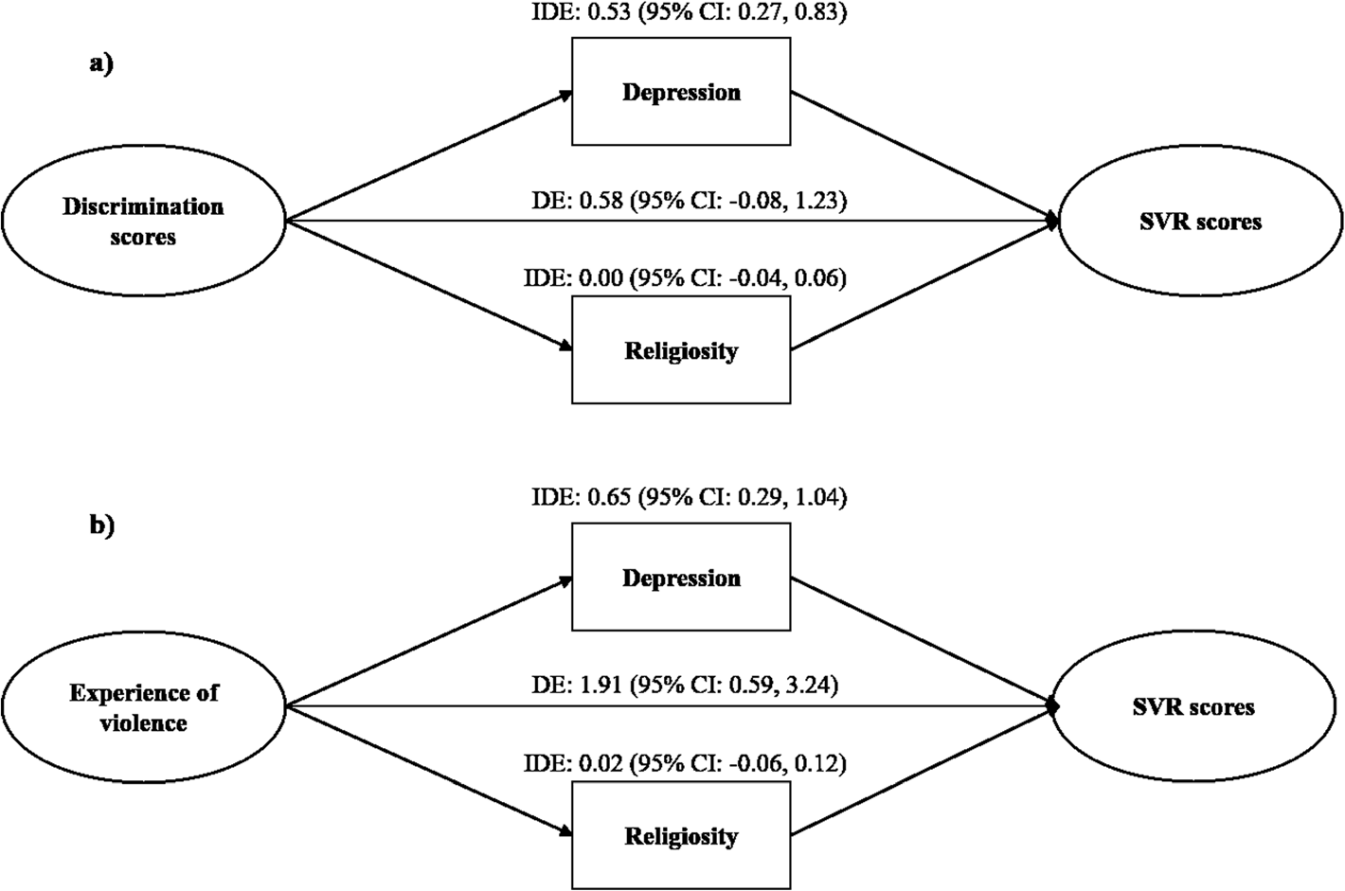
Graphical representation of mediation analyses. Legend: Direct and indirect effects of **a** discrimination and **b** experience of violence on sympathy for violent radicalization scores

Results from mediation analyses examining the pathways of the associations between ExV, discrimination, and SVR scores showed that depression, but not religiosity, was a significant and important mediator (Table 4 and Fig. 1).

**Table 4:** Results from mediation analyses (*n* = 1190)

Exposure	Mediator	Effect decomposition	Estimate	95% CI	*P*-value
Discrimination (continuous score)	Depression	ACME	0.53	0.27, 0.83	< 0.001
		ADE	0.58	−0.08, 1.23	0.12
		TE	1.12	0.50, 1.72	< 0.001
		Proportion mediated	0.47	0.20, 1.15	< 0.001
	Religiosity	ACME	0.00	−0.04, 0.06	0.86
		ADE	1.14	0.50, 1.80	< 0.001
		TE	1.14	0.50, 1.80	< 0.001
		Proportion mediated	0.00	−0.05, 0.06	0.86
Discrimination (Dichotomous; < / ≥ 1 event)	Depression	ACME	0.82	0.40, 1.29	< 0.001
		ADE	1.36	−0.06, 2.68	0.06
		TE	2.17	0.77, 3.47	< 0.001
		Proportion mediated	0.37	0.16, 1.00	< 0.001
	Religiosity	ACME	0.03	−0.08, 0.16	0.62
		ADE	2.17	0.84, 3.52	< 0.001
		TE	2.20	0.91, 3.60	< 0.001
		Proportion mediated	0.01	−0.04, 0.10	0.62
Experience of Violence	Depression	ACME	0.65	0.29, 1.04	< 0.001
		ADE	1.91	0.59, 3.24	< 0.001
		TE	2.56	1.21, 3.83	< 0.001
		Proportion mediated	0.25	0.10, 0.57	< 0.001
	Religiosity	ACME	0.02	−0.06, 0.12	0.62
		ADE	2.54	1.38, 3.76	< 0.001
		TE	2.56	1.38, 3.76	< 0.001
		Proportion mediated	0.01	−0.03, 0.05	0.62

For instance, depression scores accounted for 47 and 25% of the total effect between discrimination and ExV and SVR scores, respectively. When considering the dichotomous scores of discrimination, we observed the same pattern with 37% of the total effect on SVR scores mediated by depression. Results regarding the conditional direct effect corresponding to the effect of exposures on SVR scores if we were to intervene on depression and keep all students below the clinical cutoff of 1.75 showed a CDE of 0.80 (95% CI: 0.14, 1.47) for a 1-SD increase in discrimination scores. Likewise, the CDE of experiencing at least one discriminating event was 1.65 (95% CI: 0.29, 3.02), whereas the CDE of experiencing violence was 2.14 (95% CI: 0.87, 3.41).

### Sensitivity analyses

Results using imputed datasets and analyses using the Radicalism Intention Scale (RIS) instead of the scores from the Sympathies for Radicalization scale yielded similar patterns (see Additional file 1).

## Discussion

The present study adopts an ecological framework to investigate the potential moderating or mediating role of depression, religiosity and social support in the expected association between social adversity (i.e., perceived discrimination and exposure to violence) and SVR in a sample of college students in Quebec (Canada). Overall, the levels of SVR in our sample were low. In addition, gender and age were associated with SVR, confirming the classical predominance of SVR in males and in younger (19–24 y.o) youth [18, 48]. The fact that SVR was significantly higher in students without a declared religion and lower in first generation immigrants shatters some of the popular beliefs about the association between religion, immigration and SVR. These results partially coincide with those of Coid et al., [21] and Ellis et al., [13], who found support for extremism both in minorities and the majority, although under different forms, and with Pauwels et al. [9] who emphasized the relatively unrecognized importance of majority extremism. The distribution of SVR in this general population sample of students confirms that violent radicalization should be understood as a systemic phenomenon, affecting a society as a whole [48]. Although levels of SVR in our sample were low, our results show concerning levels of distress and social adversity among students, supporting the hypothesized link between social adversity, distress and SVR. The fact that youth participants reported that the majority of discrimination events took place in their education institutions indicates that the efforts to address bullying, intimidation and discrimination in schools and colleges should probably be intensified and considered as an important component of these programs.

### Social adversity and SVR

Results confirm the significant associations between exposure to violence, discrimination and higher levels of SVR. The role of exposure to violence and trauma as a contributor to the onset of delinquent and criminal behaviors is well established [49, 50]. Experiences of trauma and abuse appear to be factors which, in combination with other social processes, contribute both to actual violent extremism behaviors [18, 51] and to SVR [13].

The association between discrimination and externalizing behaviors is also well documented in youth [52, 53]. Discrimination has been shown to have cumulative effects with childhood trauma experiences and both are increasingly associated to paths to violent radicalization. Our results again coincide with Pauwels and DeWaele [9]. Taken together, the effects of exposure to violence and discrimination on SVR invite to take more into account the impact of such human perpetrated adversities in the content and development of violent radicalization prevention programs. It is to be noted, however, that the dominance of ambiguous forms of discrimination events illustrates the importance of micro-aggressions in the youth’s lives. These are often missed or minimized by school administrations eager to protect the image of their institutions and are often difficult to address.

### The mediating role of depression

Students in our sample reported very high levels of anxiety and depression. However, only depression scores were significantly associated with SVR suggesting some specificity in the symptom profile and mental health variables associated with SVR. These results are in line with the body of studies associating depressive symptoms with violence and aggressive manifestations, and replicate, in the Quebec context, Bhui’s findings [19, 20] on the direct relation between depression symptoms and SVR in the UK. Unlike findings observed by Bhui (10) who examined the impact of non-violent adverse life events on SVR and found no significant mediating effect, our results further contribute to this literature by showing that depression also acts as a significant mediator of the effect of exposure to violence and discrimination on SVR, suggesting that a significant part of the effect of social adversity on SVR operates through depressive symptoms and associated anger and rage. This finding offers opportunities to tackle this issue by intervening on the mediator (i.e. depression). Indeed, the conditional direct effect analysis suggests that treating depression would significantly decrease SVR, decreasing not only the direct effect of depression but also a part of the effect of discrimination and violence on SVR (~ 20%). This result certainly calls for the integration of mental health and psychosocial services in proximity environments (such as on premises in schools and colleges) in order to provide support to depressed youth who are also undergoing social adversity (social violence and discrimination) as this may make them vulnerable to SVR.

### The moderating role of social support and religiosity

In this study both social support and religiosity moderated the negative effect of social adversity on SVR. In the literature social support has been shown to be a central, yet complex, factor interacting with SVR [54], and our results confirm that social support buffers the link between social adversity and SVR in a sample of both majority and minority students.

Our results provide some evidence on the protective role of religiosity in the relation between social adversity and SVR in both majority and minority student samples, and converge with those of Simon et al. [26], who showed that stronger religious identification was associated with lower sympathy for radical action in a sample of immigrants in Germany. Religiosity was not found to mediate the link between social adversity and SVR, suggesting that it is not influenced by discriminatory and violence experiences. The religiosity revival among many young Muslims worldwide may constitute a protective coping strategy in the form of a new self-chosen identity in the face of challenges of living in a society they perceive as hostile to their ethnic or religious origins [55–57]. The discrepancies in studies around the role of religiosity may indicate that religiosity can simultaneously be protective for a majority of youth while it may also sometimes become a risk factor for some when there is adhesion to religious knowledge provided through Internet or through radicalized peers or mentors [56]. More research is needed to shed light on this possibility. Nonetheless, our findings suggest that the potential protective role of religiosity should be considered both in clinical intervention and in prevention programs.

### Limitations

This study has some limitations which need to be mentioned. First, we used a cross-sectional design which prevents us from drawing any conclusions about causality. Longitudinal studies are needed to shed light on the developmental trajectories of the individual and societal factors involved in students’ SVR. Second, the online questionnaire method of recruitment does not provide a profile of non-responders and is associated with a wide variation in response rates, which is delicate to interpret because of the number of possible associated factors that may result in selection bias. This important bias is inherent to on-line surveys on wide population samples. However, students' open ended comments confirmed that the online questionnaire facilitated the participation of those who would not have accepted a phone or face-to-face interview because the sensitivity of the topic. Third, the missing data is another limitation, although the sensitivity analyses suggest that this has not altered the observed patterns of associations. Fourth, our sample may not be representative of young people of different ages and who are not attending college. Indeed, a lower level of education is usually associated with less nuanced worldviews, which in turn have been associated with higher cognitive radicalization [58]. However, a high number of young people attend school and colleges, which have been reported as important radicalization vectors and recruitment sites [59], suggesting that schools and colleges may play a key role also in terms of prevention efforts [60]. Also, our findings showed that second- and third-generation (and above) immigrant students had similar SVR scores, reporting higher scores than first-generation immigrant students. Although we controlled for immigrant status (i.e., first-generation, second-generation and third-generation and above) in all our analyses, future studies should further explore the commonalities and differences between immigrant and non-immigrant students in the associations of risk (e.g., depression, discrimination, age) and protective factors (e.g., social support, religiosity) with SVR, taking generational status into account. Another limitation is the use of mediation analyses that account for a single mediator at a time. Further studies should account for all potential mediators simultaneously using recent methodological developments in mediation analyses, especially when these mediators impact each other [61]. However, our two mediators, i.e. religiosity and depression, had a weak correlation (Pearson *ρ* = 0.05) and this suggests that a single mediator at a time approach may still provide valid findings. Finally, it is worth mentioning that the observed effect sizes in this study are relatively modest and may not be indicative of any positive attitude towards violent radicalization. However, in the context of populations, the impact of a factor at the population level depends not only on the magnitude of its impact, or its effect size, but also on the distribution of the exposure factor. Given the widespread and ubiquitous exposure to both exposures, i.e. 44% exposed to violence and 38% exposed to discrimination, these small effect sizes may have a considerable impact at the population level [62].

## Conclusions

In spite of these limitations, the results represent the first source of local data on SVR in youth in Quebec and in Canada and provide important indications to develop prevention programs in college settings. First, the association of social adversity with SVR confirms the importance to target discrimination and bullying, as new polarized manifestations of social conflict, in prevention programs in schools and colleges. The predominance of micro- aggressions requires to go beyond the usual anti-bullying policies and to integrate strategies aiming at increasing the awareness of the other and at reflecting on diversity and identity in school classes and in other school-based activities. Programs promoting inclusion, equity and diversity should be a priority, always keeping in mind their adaptation to the local context and the institutional dynamics. Second, the mediating effect of depression emphasizes the importance of developing psychosocial support in proximity services to address the distress and anger of youth who have been exposed to different forms of human violence and discrimination. Presently, student services in colleges are not offering a lot of support to youth, and the available support is often given mainly to students who attract the attention of the staff. Advertising clinical services for trauma and for depression, and facilitating the access to such services, would certainly decrease the despair in students, as well as their anger, which can otherwise be expressed through social media hate discourses. Finally the protective roles of religiosity and social support indicate that policy makers and program developers may need to support programs that foster social cohesion and enhance youth and community resilience. With regards to religion, this is a real challenge in Quebec given that the majority has a bitter sour historical experience of religion which presently interacts with the common anti-muslim prejudices and the world upsurge in anti-semitism. Colleges have to reflect on the place of religion in their institutions in order to overcome this majority–minority divide and promote respect based on a Human Rights approach. In line with the World Health Organization recommendations for the prevention of violence [63], such results support the importance of adopting an ecological and public health approach to the study of violent radicalization phenomena, able to take into account the interplay of individual, contextual and social variables in determining the risks associated with SVR, while focusing on prevention.

## Supplementary information

**Additional file 1.** Sensitivity analyses. Results of sensitivity analyses using imputed datasets and analyses using the Radicalism Intention Scale (RIS) instead of the scores from the Sympathies for Radicalization scale.

## Data Availability

Given the strong risk of subject and institution identification within a critical context, we cannot deposit our datasets on a publicly available repository. However, we will work on de-identifying all the data, and make them available for other researchers upon request.

## Abbreviations

ExV: Exposure to Violence
SVR: Sympathy for Violent Radicalization
