# Supplementary material for: From social adversity to sympathy for violent radicalization: the role of depression, religiosity and social support
Source: Arch Public Health. 2019 Oct 25;77:45. doi: 10.1186/s13690-019-0372-y (PMC6813961; doi:10.1186/s13690-019-0372-y)
Supplement: Supplementary file 1 — Additional file 1. Sensitivity analyses. Results of sensitivity analyses using imputed datasets and analyses using the Radicalism Intention Scale (RIS) instead of the scores from the Sympathies for Radicalization scale. [file 13690_2019_372_MOESM1_ESM.docx]

**Additional file 1**

**Sensitivity analyses with imputation (m=10)**

| Exposure | Mediator | Effect decomposition | Estimate | 95% CI |
| --- | --- | --- | --- | --- |
| Discrimination | Depression | ACME | 0.38 | 0.15, 0.62 |
|  |  | ADE | 0.56 | -0.03, 1.16 |
|  |  | TE | 0.94 | 0.41, 1.52 |
|  |  | Proportion mediated | 0.85 | 0.12, 1.08 |
|  | Religiosity | ACME | 0.01 | -0.04, 0.06 |
|  |  | ADE | 0.95 | 0.34, 1.51 |
|  |  | TE | 0.96 | 0.36, 1.52 |
|  |  | Proportion mediated | 0.01 | -0.04, 0.09 |
| Discrimination (Dichotomous; < / ≥ 1 event) | Depression | ACME | 0.63 | 0.29, 1.03 |
|  |  | ADE | 1.10 | -0.10, 2.39 |
|  |  | TE | 1.74 | 0.65, 2.96 |
|  |  | Proportion mediated | 0.46 | 0.14, 1.14 |
|  | Religiosity | ACME | 0.02 | -0.07, 0.12 |
|  |  | ADE | 1.75 | 0.52, 2.77 |
|  |  | TE | 1.77 | 0.55, 2.82 |
|  |  | Proportion mediated | 0.02 | -0.03, 0.10 |
| Experience of Violence | Depression | ACME | 0.54 | 0.25, 0.88 |
|  |  | ADE | 1.71 | 0.55, 2.87 |
|  |  | TE | 2.25 | 1.12, 3.39 |
|  |  | Proportion mediated | 0.26 | 0.10, 0.55 |
|  | Religiosity | ACME | 0.01 | -0.04, 0.10 |
|  |  | ADE | 2.18 | 1.09, 3.28 |
|  |  | TE | 2.19 | 1.10, 3.29 |
|  |  | Proportion mediated | 0.01 | -0.02, 0.05 |

**Sensitivity analyses with Total RIS**

| Exposure | Mediator | Effect decomposition | Estimate | 95% CI |
| --- | --- | --- | --- | --- |
| Discrimination | Depression | ACME | 0.22 | 0.05, 0.40 |
|  |  | ADE | 0.19 | -0.25, 0.58 |
|  |  | TE | 0.41 | 0.03, 0.76 |
|  |  | Proportion mediated | 0.51 | 0.06, 3.11 |
|  | Religiosity | ACME | 0.01 | -0.02, 0.04 |
|  |  | ADE | 0.36 | -0.02, 0.76 |
|  |  | TE | 0.37 | -0.02, 0.76 |
|  |  | Proportion mediated | 0.01 | -0.08, 0.27 |
| Discrimination (Dichotomous; < / ≥ 1 event) | Depression | ACME | 0.39 | 0.15, 0.65 |
|  |  | ADE | 0.01 | -0.81, 0.87 |
|  |  | TE | 0.39 | -0.43, 1.22 |
|  |  | Proportion mediated | 0.66 | -7.92, 10.21 |
|  | Religiosity | ACME | 0.01 | -0.05, 0.10 |
|  |  | ADE | 0.37 | -0.37, 1.13 |
|  |  | TE | 0.38 | -0.35, 1.15 |
|  |  | Proportion mediated | 0.02 | -0.63, 0.64 |
| Experience of Violence | Depression | ACME | 0.28 | 0.07, 0.53 |
|  |  | ADE | 0.67 | 0.09, 1.41 |
|  |  | TE | 0.96 | 0.21, 1.64 |
|  |  | Proportion mediated | 0.29 | 0.07, 1.19 |
|  | Religiosity | ACME | 0.01 | -0.03, 0.08 |
|  |  | ADE | 0.93 | 0.21, 1.66 |
|  |  | TE | 0.94 | 0.21, 1.68 |
|  |  | Proportion mediated | 0.01 | -0.06, 0.12 |
